# Myostatin genetic inactivation inhibits myogenesis by muscle-derived stem cells *in vitro *but not when implanted in the mdx mouse muscle

**DOI:** 10.1186/scrt152

**Published:** 2013-01-07

**Authors:** James Tsao, Dolores A Vernet, Robert Gelfand, Istvan Kovanecz, Gaby Nolazco, Kevin W Bruhn, Nestor F Gonzalez-Cadavid

**Affiliations:** 1 Department of Internal Medicine, Charles Drew University (CDU), 1731 East 120th Street, Los Angeles, CA 90059, USA; 2 Department of Urology, David Geffen School of Medicine at UCLA, 10833 Le Conte Avenue, Los Angeles, CA 90095, USA; 3 Los Angeles Biomedical Research Institute (LABioMed) at Harbor-UCLA Medical Center, 1124 West Carson Street, Torrance, CA 90502, USA

## Abstract

**Introduction:**

Stimulating the commitment of implanted dystrophin+ muscle-derived stem cells (MDSCs) into myogenic, as opposed to lipofibrogenic lineages, is a promising therapeutic strategy for Duchenne muscular dystrophy (DMD).

**Methods:**

To examine whether counteracting myostatin, a negative regulator of muscle mass and a pro-lipofibrotic factor, would help this process, we compared the *in vitro *myogenic and fibrogenic capacity of MDSCs from wild-type (WT) and myostatin knockout (Mst KO) mice under various modulators, the expression of key stem cell and myogenic genes, and the capacity of these MDSCs to repair the injured gastrocnemius in aged dystrophic mdx mice with exacerbated lipofibrosis.

**Results:**

Surprisingly, the potent *in vitro *myotube formation by WT MDSCs was refractory to modulators of myostatin expression or activity, and the Mst KO MDSCs failed to form myotubes under various conditions, despite both MDSC expressing Oct 4 and various stem cell genes and differentiating into nonmyogenic lineages. The genetic inactivation of myostatin in MDSCs was associated with silencing of critical genes for early myogenesis (*Actc1, Acta1, and MyoD*). WT MDSCs implanted into the injured gastrocnemius of aged mdx mice significantly improved myofiber repair and reduced fat deposition and, to a lesser extent, fibrosis. In contrast to their *in vitro *behavior, Mst KO MDSCs *in vivo *also significantly improved myofiber repair, but had few effects on lipofibrotic degeneration.

**Conclusions:**

Although WT MDSCs are very myogenic in culture and stimulate muscle repair after injury in the aged mdx mouse, myostatin genetic inactivation blocks myotube formation *in vitro*, but the myogenic capacity is recovered *in vivo *under the influence of the myostatin+ host-tissue environment, presumably by reactivation of key genes originally silenced in the Mst KO MDSCs.

## Introduction

The lipofibrotic degeneration of skeletal muscle (that is, excessive deposition of endomysial collagen, other extracellular matrix, and fat), characterizes muscle dystrophy, and in particular Duchenne muscular dystrophy (DMD) [1,2], as seen also in its animal model, the mdx mouse [3-5]. This process, associated with inflammation and oxidative stress [6], is partially responsible for the severe muscle contractile dysfunction in DMD and the mdx mouse, caused mainly by the bouts of myofiber necrosis due to dystrophin genetic inactivation. In the gastrocnemius, these processes are rather mild in young animals but become particularly severe after 8 to 10 months of age [4]. Dystrophic muscle fibrosis not only is a major factor for DMD mortality, but also hampers the uptake and survival of cells implanted for potential therapeutic approaches [7] and/or may drive their differentiation into myofibroblasts [4]. Therefore, trying to ameliorate this process while stimulating myogenesis constitutes an ancillary strategy to favor repair and regeneration of dystrophic muscle tissue, even under ineffective or absent dystrophin replacement.

Although pharmacologic approaches to combat muscle lipofibrotic degeneration and the underlying chronic inflammation are being widely investigated, biologic factors such as myostatin, the main negative regulator of muscle mass [8], are also potential key targets. Myostatin, a member of the TGF-β family, aggravates muscle dystrophy not only as an antimyogenic agent but also as a profibrotic and adipogenic factor [9-14]. Inhibition of myostatin by using its propeptide, shRNA, or specific antibodies, improves myogenesis and reduces fibrosis in the mdx mouse [15-17] and also in the rat [18]. The same effects are generated in response to genetic deletion of myostatin in the myostatin knockout (MST KO) mouse, in which myofiber hypertrophy is associated with less fat and reduced fibrosis [19-23].

It is assumed that in the dystrophic or injured muscle, tissue repair and the opposite process of lipofibrotic degeneration involve not only the differentiation of progenitor satellite cells and fibroblasts into myofibers and myofibroblasts, respectively, but also the modulation of lineage commitment by stem cells present in the adult muscle [24-26]. These stem cells have been isolated from the rodent and human skeletal muscle and named, in general, muscle-derived stem cells (MDSCs), because they have the ability to differentiate *in vitro *into multiple cell lineages and to generate myofibers, osteoblasts, cardiomyocytes, or smooth muscle cells after implantation into the skeletal muscle, bone, heart, corpora cavernosa, or vagina, respectively [27-31]. They are not satellite cells and may act also by secreting paracrine growth factors that are believed to modulate the differentiation of endogenous stem cells or the survival of differentiated cells in the tissue [32-34]. However, the roles of MDSCs in the biology and pathophysiology of the skeletal muscle are largely unknown.

Myostatin modulates the differentiation of pluripotent cells *in vitro*, albeit in some cases, with conflicting outcomes [14,35-37]. It also inhibits the proliferation and early differentiation of both satellite cells from the skeletal muscle and cultured myoblasts, and blocking its expression improves the success of their *in vivo *transplantation [38-40]. To our knowledge, no reports are available on myostatin effects on MDSC differentiation, either *in vitro *or in the context of repairing the exacerbated lipofibrosis in the injured muscle of aged mdx mice.

MDSCs obtained from wild-type (WT) mice have been tested experimentally, aiming to trigger repair of the mdx muscle with variable results [31-45], but they appear to be superior in this respect to myoblasts or satellite cells [46]. However, some of the main limitations of myoblast therapy, when translated from the murine models into DMD and other human muscle dystrophies, may also affect the MDSCs and other types of stem cells [47]. Therefore, it is a therapeutic goal to enhance the repair capacity of WT MDSCs by *in vitro *or *in vivo *modulation of their multilineage potential, and to stimulate or even awake endogenous stem cells of dystrophic muscle to regenerate myofibers while avoiding differentiation into cells responsible for lipofibrotic degeneration. Such an approach may be provided by the use of MDSCs where myostatin is genetically inactivated (that is, obtained from the Mst KO mouse), under the assumption that myogenesis would be stimulated and the undesired lineage commitment reduced, even when implanted into a host tissue environment with normal myostatin expression. No reports are available on the *in vitro *and *in vivo *differentiation of these MDSCs and how this affects, even paracrinely, muscle repair.

### Potential *in vitro *modulation of MDSCs, or the effects that myostatin or dystrophin gene inactivation exert on this balance

In the current study, we have investigated the *in vitro *myogenic versus fibrogenic and adipogenic differentiation of Mst KO MDSCs *vis-à-vis *the WT counterpart, and the effects of manipulation of these processes by modulating myostatin expression or activity, and by other putative regulators of muscle mass and fibrosis. Their differential *in vitro *features in terms of the expression of some key stem cell and myogenic genes, and the repair ability of Mst KO MDSCs in the injured mdx muscle, also were studied. The ultimate goal is to gain a preliminary insight into how *in vitro *preconditioning of MDSCs by pharmacologic or gain-of-function approaches may modulate their capacity to repair dystrophic skeletal muscle, to design *in vivo *pharmacologic interventions that may mimic these processes, and even myostatin blockade in the host muscle to activate myogenesis in the endogenous dystrophin-negative MDSCs.

## Materials and methods

## MDSC isolation

Mst knockout mice (C57BL/6J/Mst^-/-^), referred to here as Mst KO, are regularly maintained and bred in our vivarium on a BL/6 background [48], derived from the original strain on a Balb/c background. Aged-matched wild-type control mice (C57BL/6J), referred to here as WT, were from Jackson Laboratories (Bar Harbor, ME, USA). Hindlimb muscles from the WT and Mst KO male mice (12 to 16 weeks old) were subjected to the preplating procedure to isolate MDSCs [49,50], by using a modification of a well-validated method that has led to extensively characterized stem cell populations [5,27-30,46,49]. Tissues were dissociated by using sequentially collagenase XI, dispase II, and trypsin, and after filtration through 60-μm nylon mesh and pelleting, the cells were suspended in plating medium (PM), containing Dulbecco Modified Eagle Medium (DMEM), with 10% fetal bovine serum (FBS), 10% horse serum, and 0.5% chick embryo extract (US Biological, Marblehead, MA, USA). Cells were plated onto collagen I-coated flasks for 1 hour (preplate 1 or pP1), and 2 hours (preplate 2 for pP2), followed by sequential daily transfers of nonadherent cells and replatings for 2 to 6 days, until preplate 6 (pP6). The latter is the cell population containing MDSCs. Sca1^+ ^cells were selected with immunobeads (Milteni, Auburn, CA, USA) coated with antibody against Sca1 as small cells with a large nucleus that easily form clusters/spheroids [24-27]. Cells were subjected to flow cytometry, as described later, for the MDSC standard markers Sca1, CD34, and CD44, and for the key stem-cell gene, Oct 4 [43], maintained in growth medium (GM) GM-20 (DMEM, with 20% FBS) on regular culture flasks (no coating) and used in passages 14 to 28. WT MDSCs have been maintained in our laboratory for at least 40 generations with the same, or even increasing, growth rate.

## Flow cytometry

MDSC and KO cells were grown in GM-20, washed twice with Hanks, disaggregated by repeated pipetting in Cell Stripper (Mediatech, Manassas, VA, USA), pelleted, and resuspended in staining buffer consisting of PBS, 3% FBS, 0.01% Na azide (SB). Cells were incubated in the presence of antibodies for 30 minutes on ice, washed twice with SB, and finally resuspended in SB for flow cytometry on an LSR II (BD Biosciences, San Jose, CA, USA). Data analysis and plotting were done by using FACSDiva Version 6.1.1 software. All fluorophore-conjugated antibodies and isotype controls were from eBioscience (San Diego, CA, USA), as follows: CD44-APC-eFluor 780; CD34-eFluor 660; Sca1-PE; Oct 4-PE (performed separately, after cell permeabilization with BD CytoFix/CytoPerm Kit), and the appropriate rat isotype controls IgG2b-APC-eFluor 780, IgG2a-eFluor 660, and IgG2a-PE. BD CompBeads (rat) were used for compensation.

## Stem cell characterization, differentiation, and modulation

MDSC cultures were analyzed for the expression of stem cell markers, as described later, on collagen-coated six-well plates and eight removable-chamber plates. Multipotency was analyzed in 2-week incubations with GM-20 or GM-10 (GM with 10% FBS) supplemented or not with 10 n*M *DMSO or 5 ng/ml TGF-β1, or, to induce myofiber formation, after reaching confluence, for 2 to 3 weeks with GM-HC (DMEM, 10% FBS, 5% horse serum, and 50 μm hydrocortisone to promote proliferation, a key event in myogenic differentiation) [44,45], or as described. In certain cases, cultures were treated with or without 20 μ*M *5'-azacytidine (AZCT) in GM-20 for 3 days to induce multipotency, before switching them to the appropriate medium [11,14,51].

For the tests on the modulation of MDSCs skeletal myotube formation by various factors, cells were allowed to reach confluence, switched to GM-HC, and incubated for 2 weeks with 2 μg/ml recombinant 113-amino acid myostatin protein (R-Mst), a biologically active recombinant 16-kDa protein containing 113 amino acid residues of the processed human myostatin protein (BioVendor Laboratory Medicine Inc., Palackeho, Czech Republic) [14,52,53], or with a recombinant mouse follistatin protein (RD Systems, Minneapolis, MN, USA) at 0.2 μg/ml [11,14], changing medium twice a week. In other experiments, incubations with the monoclonal (Chemicon International, Temecula, CA, USA) and polyclonal (Millipore Corp, Billerica, MA, USA) antibodies against myostatin (1:20) were substituted for the previous treatments. Alternatively, the adenoviruses expressing the mouse myostatin full-length cDNA under the CMV promoter (AdV-CMV-Mst375) and an shRNA, which targets myostatin RNA and inhibits more than 95% of myostatin gene expression [11,14,18] (AdV-Mst shRNA) were transduced into MDSCs at 80% confluence. Then cells were switched to GM-HC medium, as described earlier.

## Implantation of MDSCs into skeletal muscle

Male mdx mice (C57BL/6/10ScSn-Dmd^mdx^), referred to here as "mdx", obtained from Jackson Laboratories (Bar Harbor, ME, USA) were allowed to reach 10 months of age, to allow lipofibrotic degeneration to become more evident, not only in the diaphragm but also in the gastrocnemius. In contrast, in young animals (12 to 16 weeks of age), the first round of muscle necrosis and regeneration had already subsided (stable phase).

Mice were treated according to National Institutes of Health (NIH) regulations with an Institutional Animal Care and Use Committee-approved protocol. In one experiment, the WT and mdx MDSCs (0.5 to 1.0 × 10^6 ^cells/50 μl saline) were labeled with the nuclear fluorescent stain, 4',6-diamidino-2-phenylindole (DAPI) [27-30], and implanted aseptically under anesthesia into the surgically exposed tibialis anterior. The muscle had been cryoinjured by pinching it for 10 seconds with a forceps cooled in liquid nitrogen immediately before implantation. Control mice with the same cryoinjury received saline. Mice were killed after 2 weeks, and the tibialis excised and subjected to cryoprotection in 30% sucrose, embedding in OCT, and cryosectioning.

In another experiment, the DAPI-labeled WT and Mst KO MDSCs (0.5 × 10^6 ^cells/50 μl GM) were implanted into the central region of the surgically exposed left gastrocnemius of 10-month-old mdx mice, which 4 days earlier had been injured with two injections of notexin in both tips of the muscle (total: 0.2 μg in 10 μl saline). Control muscle-injured mice were injected with saline (*n *= 5/group). Mice were killed at 3 weeks, the gastrocnemius excised, and a section around the site of notexin injection was used for cryosectioning. The remaining tissue was kept frozen at -80°C.

### Immunocytochemistry and dual immunofluorescence

Cells on collagen-coated eight-well removable chambers, fixed in 2% *p*-formaldehyde, and 10 μm unfixed frozen tissue sections, were reacted [10,11,14,18,29,30] with some of the following primary antibodies against (a) human myosin heavy-chain fast, detecting both MHC-IIa and MHC-IIb); monoclonal, 1:200 Vector Laboratories, Burlingame, CA, USA), a marker for skeletal myotubes and myofibers; (b) human ASMA (mouse monoclonal in Sigma kit, 1:2, Sigma Chemical, St. Louis, MO, USA), a marker for both SMCs and myofibroblasts; (c) neurofilament 70 (NF70; mouse monoclonal, 1:10, Millipore); (d) Dystrophin (rabbit polyclonal, 1:200 Abcam, Cambridge, Massachusetts, USA); (e) Sca-1 (mouse monoclonal, 1:100, BD Pharmingen, San Jose, CA, USA) and M.O.M blocking kit (Vector, Burlingame, CA, USA); and (f) Oct 4 (rabbit polyclonal, 1:500, BioVision, Mountain View, CA, USA). When MDSCs in eight-well chambers were not previously tagged with DAPI, all nuclei were stained with coverslips with DAPI antifading emulsion.

Cultures or tissue sections not involving DAPI labeling were subjected to immunohistochemical detection by quenching in 0.3% H_2_O_2_, blocking with goat (or corresponding serum), and incubated overnight at 4°C with the primary antibody. This was followed by biotinylated anti-mouse IgG (Vector Laboratories), respectively, for 30 minutes, the ABC complex containing avidin-linked horseradish peroxidase (1:100; Vector Laboratories), 3,3' diaminobenzidine, and counterstaining with hematoxylin, or no counterstaining. For cells labeled with DAPI, fluorescent detection techniques were used. The secondary anti-mouse IgG antibody was biotinylated (goat, 1:200, Vector Laboratories), and this complex was detected with streptavidin-Texas Red. After washing with PBS, the sections were mounted with Prolong antifade (Molecular Probes, Carlsbad, CA, USA). Negative controls in all cases omitted the first antibodies or were replaced by IgG isotype. In the case of Oct 4, streptavidin-FITC was used.

In tissue cryosections for experiments involving DAPI-labeled cells (10 μm), tissue sections were processed in regions where the DAPI^+ ^cells could be detected. Muscle fibers were either stained with hematoxylin/eosin, or by MHC-II antibody, either by Texas red fluorescence as previously described, or with the diaminobenzidine tetrahydrochloride-based detection method (Vectastain-Elite ABC kit; Vector Labs), counterstaining with Harris hematoxylin. Tissue sections that were incubated with mouse IgG instead of the primary antibody served as negative controls. The sections were viewed under an Olympus BH2 fluorescent microscope, and cell cultures, under an inverted microscope. In some cases, the cytochemical staining was quantitated by image analysis by using ImagePro-Plus 5.1 software (Media Cybernetics, Silver Spring, MD, USA) coupled to a Leica digital microscope bright-field light fluorescence microscope/VCC video camera. After images were calibrated for background lighting, integrated optical density (IOD, area × average intensity) was calculated.

## Gene transcriptional expression profiles

Pools of total cellular RNA from three T25 flasks for each MDSC cultured in DM-20 were isolated with Trizol-Reagent (Invitrogen, Grand Island, NY, USA) and subjected to DNAse treatment, assessing RNA quality by agarose gel electrophoresis. cDNA gene microarrays (SuperArray BioScience Corp., Frederick, MD, USA) [11,24,41] were applied, by using the mouse stem cell (OMM-405), Oligo GEArray microarray. Biotin-labeled cDNA probes were synthesized from total RNA, denatured, and hybridized overnight at 60°C in GEHybridization solution to these membranes. Chemiluminescent analysis was performed per the manufacturer's instructions. Raw data were analyzed by using GEArray Expression Analysis Suite (SuperArray BioScience Corp.). Expression values for each gene based on spot intensity were subjected to background correction and normalization with housekeeping genes, and then fold changes in relative gene expression were calculated. Microarray data were deposited in the Gene Expression Omnibus (GEO) public repository (accession number GSE28986).

The expression of some of the down- or upregulated genes detected earlier was examined on 1 μg RNA isolated from consecutive similar incubations performed in triplicate by reverse transcription (RT) by using a 16-mer oligo(dT) primer, as previously described [11,27], and the resulting cDNA was amplified using PCR in a total volume of 20 μl. The locations of the primers used for the quantitative estimation of mouse myostatin mRNA were nts 136 to 156 (forward) and 648 to 667 (reverse), numbering from the translation initiation codon, as previously described. For mouse GAPDH primers, sequences were from the mRNA sequence NM_008084.2, with a forward primer spanning nts 778-797 and reverse primer spanning nts 875-852, with a product length of 98 nt.

Additional primers were designed by using the NCBI Primer Blast program applied to mRNA sequences and synthesized by Sigma-Aldrich. Numbering refers to the length in NT from the 5' end of the mRNA: (a) Acta1 (skeletal muscle actin) NM_009606.2 (forward 501 to 520 and reverse 841 to 822; product length, 341); (b) Actc1 (cardiac actin) NM_009608.3 (forward 38 to 58 and reverse 554 to 530, product length 517); (c) MyoD NM_010866.2 (forward 515 to 534 and reverse 1013 to 994, product length 499); and (d) Pax3 NM_008781.4 (forward 1164 to 1183 and reverse 1893 to 1874, product length 730). The number of PCR cycles used for each primer set is stated in parenthesis, as follows: Actc1 (30), Acta1 (30), MyoD1 (33), Pax3 (28), and GAPDH (26). All primers were designed to include an exon-exon junction in the forward primer, except for GAPDH and MyoD1. Negative controls omitted cDNA.

### Protein expression by Western blots

Cells were homogenized in boiling lysis buffer (1% SDS, 1 m*M *sodium orthovanadate, 10 m*M *Tris pH 7.4 and protease inhibitors, followed by centrifugation at 16,000 *g *for 5 minutes [10,11,14,18,29,30]. Then 40 μg of protein was run on 7.5% or 10% polyacrylamide gels, and submitted to transfer and immunodetection with antibodies against (a) human ASMA (monoclonal, 1:1,000; Calbiochem, Billerica, MA, USA); (b) Oct 4, as for immunohistochemistry; (c) MyoD (rabbit polyclonal 1:200, Santa Cruz Biotechnology, Dallas, TX, USA); (d) MHC (fast), as for immunohistochemistry; (e) TGF-β1 (rabbit polyclonal, 1:1,000; Promega Corporation, Madison, WI, USA); (f) myostatin (rabbit polyclonal 1:1,000; Chemicon International Inc), (g) ActRIIb (monoclonal, 1:1,000, Abcam); and (h) GAPDH (mouse monoclonal, 1:3,000, Chemicon). Membranes were incubated with secondary polyclonal horse anti-mouse or anti-rabbit IgG linked to horseradish peroxidase (1:2,000; BD Transduction Laboratories, Franklin Lakes, NJ, USA, or 1:5,000, Amersham GE, Pittsburgh, PA, USA), and bands were visualized with luminol (SuperSignal West Pico; Chemiluminescent, Pierce, Rockford, IL, USA). For the negative controls, the primary antibody was omitted.

### Statistics

Values are expressed as the mean (SEM). The normality distribution of the data was established by using the Wilk-Shapiro test. Multiple comparisons were analyzed with a single-factor ANOVA, followed by *post hoc *comparisons with the Newman-Keuls test. Differences among groups were considered statistically significant at *P *< 0.05.

## Results

### MDSC cultures from the Mst KO resemble their counterparts from WT mice in morphology, replication, cell markers, and multipotent differentiation

WT MDSCs (pP6 fraction) formed *in vitro *the most robust skeletal myotubes (see next section) at about passage 13, and WT MDSCs and Mst KO MDSCs were compared from passages 10 through 28. The morphology of the proliferating cultures was similar, but the replication times for the Mst KO MDSCs were slower than those for the WT MDSCs (27.0 versus 19.8 hours, respectively). This morphology and replication pattern continued throughout the 13- through 28-passages period of study.

**Figure 1:**
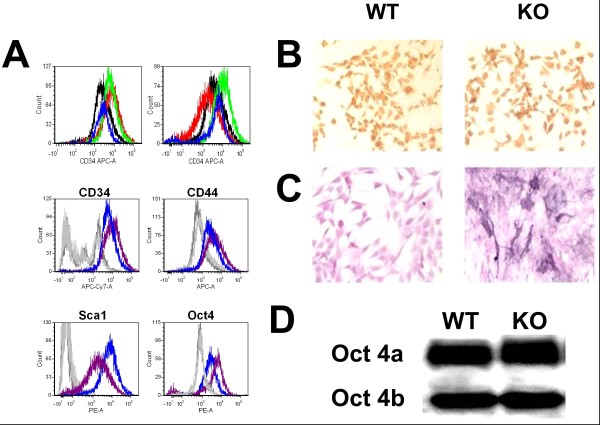
**Effect of genetic inactivation of myostatin on the expression of key stem cell marker genes in MDSC**. **(A) **Flow cytometry (no gate) was conducted for Sca1 (red), CD34 (black), CD44 (green), and Oct 4 (blue) in WT MDSCs (blue) and Mst KO MDSCs (purple), against the respective isotypes (not shown). Top panels: Left: WT MDSCs; Right: Mst KO MDSCs. Bottom panels: each antigen is compared separately for WT (blue) and Mst KO (purple), with the corresponding isotypes (WT, dark gray; Mst KO, light gray). **(B) **Representative pictures of proliferating MDSCs that were subjected to immunocytochemistry for Oct 4, showing nuclear location in most cells (200×). **(C) **Proliferating MDSCs that were subjected to cytochemistry for alkaline phosphatase (200×). **(D) **Homogenates from the same cell cultures that were subjected to Western blot for Oct 4 (nuclear Oct 4a, 45 kDa; cytoplasmic Oct 4 b, 33 kDa). WT, wild type; MDSC, muscle-derived stem cell; Mst KO, myostatin knockout.

The WT MDSC culture was previously shown to be Sca1^+ ^[28]; Sca1 selection was used for both cultures, and flow cytometry confirmed its expression in subconfluent cultures in DM-10 of both the WT and Mst KO MDSCs (Figure 1A), with negligible isotype reaction. The similarity of both types of cells was evident as well for the expression of the two MDSC markers CD34, CD44, and the key embryonic stem cell marker, Oct 4, even if the cell populations show some heterogeneity in the expression of these markers. Oct 4 in both MDSC cultures is similarly well expressed, mainly in the nuclei (the Oct 4A isoform) with some additional cytoplasmic staining (Figure 1B). That MDSCs have some embryonic stem cell features is also suggested by a mild alkaline phosphatase reaction, a feature of embryonic stem cells (Figure 1C).

The stem cell nature of the nuclear Oct 4A expression was confirmed by the detection of the 45-kDa Oct 4A transcriptionally active protein accompanied to a lesser extent by the 33-kDa Oct 4B of cytoplasmic origin (Figure 1B bottom).

**Figure 2:**
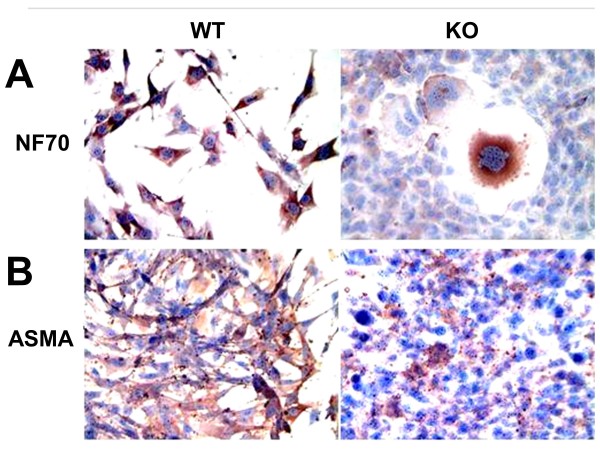
**Myostatin genetic inactivation does not block the multipotent nonmyogenic differentiation capacity of MDSCs**. Representative pictures of proliferating MDSCs treated for 2 weeks in differentiation media and subjected to immunocytochemistry for NF-70 **(A) **and ASMA **(B) **to detect marker expression of neural cells and myofibroblasts (200×). MDSC, muscle-derived stem cell; WT, wild type (muscle-derived stem cells); KO, myostatin knockout (muscle-derived stem cells); ASMA, α-smooth muscle actin.

The similarity of the Mst KO and WT MDSCs in terms of the expression of other stem cell-related genes was demonstrated by a DNA microarray analysis of a panel of 260 stem cell-related genes. Table 1 shows no substantial differences in the expression of most well-known embryonic stem cell genes, such as *c-Myc, Oct 4 (Pou5)*, alkaline phosphatase 2 and 5, telomerase reverse transcriptase, leukemia inhibitory factor (*LIF*), and mastermind-like 1, among the other related genes. This agrees with the fact that MDSCs appear to undergo a multilineage differentiation, and the capacity of these MDSCs seems to be qualitatively similar, as shown by the generation in neurogenic medium of cells expressing the neuronal marker NF70 (Figure 2), and in fibrogenic medium of cells expressing α-smooth muscle actin (ASMA), suggesting some neural or myofibroblast differentiation, respectively. However, the proportion of positive cells was lower in Mst KO MDSCs, and the cells expressing NF-70 lacked the more apparent neuronal morphology of the differentiated WT MDSCs. Both MDSC cultures also differentiated similarly into cells expressing calponin as smooth muscle cell marker and von Willebrand factor as endothelial cell marker (not shown).

**Table 1:** Some stem cell-related genes are transcribed similarly in MDSCs, irrespective of myostatin inactivation

Gene	Function	WT	KO
*Myc*	Myelocytomatosis oncogene	12.4	18.1
*Pou5F1*	Pou domain (Oct4)	10.1	16.7
*Akp 2*	Alkaline phosphatase 2	6.4	6.9
*Akp 5*	Alkaline phosphatase 5	1.2	1.6
*Tert*	Telomerase reverse transcriptase	1.0	1.0
*Utf 1*	Undifferentiated embryonic cell TP1	1.0	0.8
*Man 1*	Mastermind-like 1	13.1	16.7
*Lif*	Leukemia inhibitory factor	1.5	0.9
*PPARγ*	Peroxisome proliferator-activated receptor γ	1.1	1.8

RNA from the two MDSC types at 80% confluence (not undergoing myogenesis) were treated by DNAse and submitted to DNA microarrays. Some key stem cell genes are selected. Values are relative expression levels normalized by housekeeping genes. GAPDH expression was 98 to 102. MDSC, muscle-derived stem cell; WT, wild type; KO, myostatin knockout.

### The genetic inactivation of myostatin is, however, associated with the loss of the ability of MDSCs to form myotubes *in vitro*, and with the downregulation of key myogenic genes

**Figure 3:**
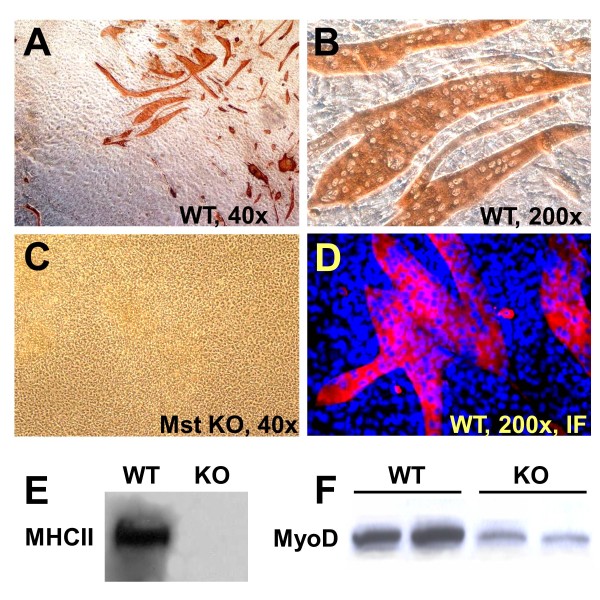
**Myostatin genetic inactivation blocks the myogenic differentiation capacity of MDSCs**. **(A-C) **Representative pictures of confluent MDSCs from the WT MDSCs and Mst KOs maintained for 2 weeks in myogenic medium and subjected to immunocytochemistry for MHC II to detect differentiation into polynucleated myotubes (magnifications as indicated). **(D) **Blue/red merge of confluent MDSCs in myogenic medium that were labeled with DAPI and submitted to immunofluorescent detection of MHC (200×); **(E) **Western blot for MHC II (210 kDa) in the confluent cultures undergoing myogenesis, and (F) for MyoD (45 kDa) in the nonconfluent cultures in nonmyogenic medium. MDSC, muscle-derived stem cell; WT, wild type; Mst KO, myostatin knockout; MHC, myosin heavy chain; DAPI: 4', 6-diaminido-phenylindole.

The WT MDSCs form large polynucleated myotubes expressing MHC II in confluent cultures on incubation for 1 to 2 weeks in GM-HC (Figure 3A, B). This myogenic medium [54,55] was selected based on its high efficiency as reported for adipose tissue stem cells and on our own preliminary results over a medium containing horse serum. However, remarkably, the Mst KO MDSC (Figure 3C) were unable to generate any myotube under these conditions, even after 4 weeks. Immunofluorescence detected high MHC II expression in the robust myotubes from WT MDSC (Figure 3D), but again, no MHC II or myotubes were found in the Mst KO confluent cultures (not shown). This is also illustrated in the Western blot analysis where the strong MHC II 210-kDa band in the WT MDSC extract is not seen in the confluent Mst KO MDSC (Figure 3E). The early myogenic marker MyoD is expressed as expected in the nonconfluent WT MDSCs in GM-20 (nonmyogenic medium), but very little in the Mst KO MDSCs.

**Figure 4:**
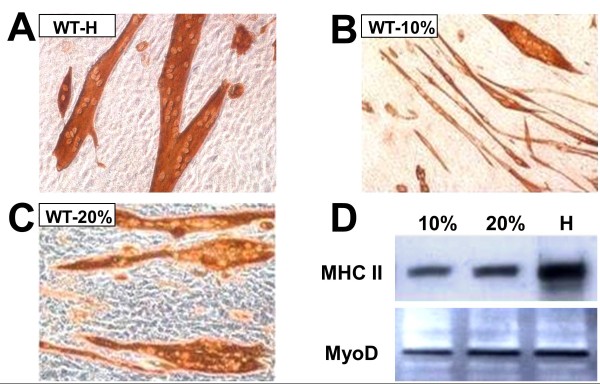
**The potent myotube-forming capacity of WT MDSCs in myogenic medium is decreased but still maintained under high serum concentrations, in the presence of steady MyoD expression**. **(A) **Representative micrographs of myotubes generated in confluent WT MDSCs maintained for 2 weeks in myogenic medium, as evidenced by immunocytochemistry for MHC II (200×); **(B) **and **(C) **as (A), but in PM with 20% or 10% serum (200×). **(D) **Representative Western blots for WT MDSCs incubated in triplicate in each of the previously mentioned cultures in the three types of media subjected to immunodetection for MHC II (210 kDa) and MyoD (44 kDa). 10, 10% PM; 20, 20% PM; H, myogenic medium; WT MDSC, wild-type (muscle-derived stem cell); MHC, myosin heavy chain; PM, plating medium.

That this was not an artifact of poor myogenesis in the GM-HC medium was shown by the fact that although robust myotube formation in the WT MDSCs occurred in GM-10 or GM-20, even if of smaller size (Figure 4B and 4C compared with Figure 4A), not a single myotube was observed with confluent Mst KO MDSCs in these media (not shown). WT MDSC myogenic differentiation in medium with a high concentration of FBS indicates that cell-to-cell contact is sufficient to trigger MDSC myogenesis, and does not require growth-factor depletion. No adipogenesis was detected by Oil red O in GM-HC medium (not shown). Western blots of parallel confluent cultures of WT MDSCs showed that MHC-II was expressed in all media (triplicate cultures), although more intensively in GM-HC (Figure 4D). No difference in MyoD expression was found among the different media.

Confluent Mst KO in several media were unable to form myotubes irrespective of passage. Myotube formation by WT MDSC cultures persisted for up to 40 passages, although the size and number of the myotubes started to decline as the passage number increased. Cultures of pP5 or pP5 from Mst KO mice obtained during the pre-plating procedure also failed to generate skeletal myotubes. Despite the drastic obliteration of MHC II^+ ^myotube formation in confluent Mst KO MDSCs, the transcriptional expression of most myogenesis-related genes in the respective proliferating cells was, as in the case of the stem cell genes in Table 1, very similar. For instance, expression of *BMPRs *(bone morphogenic protein receptors), the *Wnt *signaling receptors *frizzled *and j*ag, IGF1, Notch 1*, and *Notch 3*, was not reduced in Mst KO MDSCs as compared with the WT MDSCs (Table 2). However, six notable differences were noticed in which each gene was substantially downregulated in the Mst KO MDSCs, versus a strong expression in the WT MDSCs. They are *Spp1 *(secreted phosphoprotein 1, or osteopontin), *Actc 1 *(cardiac α-actin), *MyoD1*, *cadherin 15, Myf 5*, and *Notch 2 *(see Discussion). In contrast, other cadherins (11 and 6), related to neuromuscular development, were upregulated by ninefold and fourfold, respectively, in the Mst KO MDSCs. Other than these, a virtual 98% similarity was seen among the three MDSC types, in terms of the 260 genes investigated. An excellent correlation occurred between *MyoD *mRNA expression in both cultures and the previously detected MyoD protein levels shown in Figure 3.

**Table 2:** Some skeletal myogenesis-related genes are downregulated in MDSCs by myostatin genetic inactivation, whereas others remain unchanged

Gene	Function	WT	KO
*Spp1*	Secreted phosphoprotein 1 (osteopontin)	70.8	20.3
*Actc 1*	α-Actin (cardiac)	39.9	6.5
*Myo D1*	Myogenic differentiation 1	17.5	2.7
*Cadherin 15*	Cadherin 15	8.7	1.7
*Myf 5*	Myogenic factor 5	4.2	2.7
*Notch 2*	Notch gene homolog 2	4.2	2.8
*BMPR 2*	Bone morphogenetic receptor 2	23.3	20.3
*BMPR 1a*	Bone morphogenetic receptor 1a	8.1	10.4
*BMPR 1b*	Bone morphogenetic receptor 1b	0.8	0.8
*BMPR 4*	Bone morphogenetic protein 4	2.7	2.7
*IGF 1*	Insulin-like growth factor 1	5.1	4.2
*Jag 1*	Jagged 1	2.8	3.4
*Fzd 1*	Frizzled homolog 1	2.7	2.8
*Notch 1*	Notch gene homolog 1	2.6	2.7
*Notch 3*	Notch gene homolog 3	2.8	2.6

RNA from the two MDSC types at 80% confluence (not undergoing myogenesis) were treated with DNAse and submitted to DNA microarrays. Some key myogenesis-related genes are selected. Values are relative expression levels normalized by housekeeping genes. GAPDH expression was 98 to 102. MDSC, muscle-derived stem cell; WT, wild type; KO, myostatin knockout.

**Figure 5:**
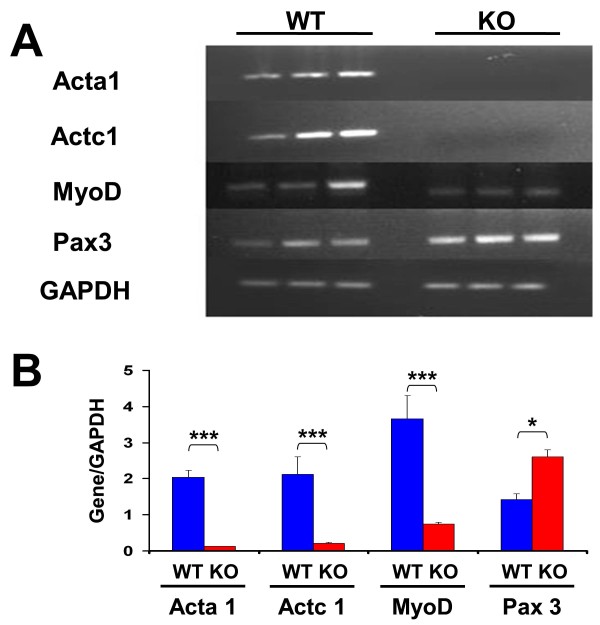
**RT-PCR confirmation of selected differences in the transcriptional expression of undifferentiated WT and Mst KO MDSCs, detected with DNA microarrays**. RNAs obtained from triplicate cultures of proliferating MDSCs, consecutive to those used for DNA microarrays in Tables 1 and 2, were subjected to RT-PCR with specific primers spanning an intron for the number of PCR cycles stated in parenthesis, as follows: Actc1 (30), Acta1 (30), MyoD1 (33), Pax3 (28), and GAPDH (26). **(A) **Ethidium bromide-stained agarose gels; **(B) **densitometry of relative band intensities referred to housekeeping gene for the indicated numbers of PCR cycles. Controls without cDNA were blank. *P < 0.05; ***P < 0.001. RT-PCR, reverse transcription polymerase chain reaction; WT, wild type; Mst KO, myostatin knockout; MDSC, muscle-derived stem cell; DNA, deoxyribonucleic acid; RNA, ribonucleic acid; GAPDH, glyceraldehyde 3-phosphate dehydrogenase.

These results were corroborated by RT-PCR for some of the mRNAs described in the tables. Figure 5A shows the gel electrophoretic pattern after staining with ethidium bromide, and Figure 5B presents the densitometric value of each band from triplicate determinations corrected by the housekeeping-gene values. These ratios are comparable between both MDSC cultures for each gene, but not among the different genes for each culture, because of the different numbers of cycles applied for the respective transcript amplification. *Actc1, Acta1*, and *MyoD *are significantly downregulated in Mst KO as compared with WT MDSCs, and *Pax 3 *is overexpressed, in good agreement with the DNA microarrays.

### Myotube formation cannot be induced in Mst KO MDSCs by stem cell-reactivating agents, and the WT MDSCs are also refractory to positive or negative modulation of myostatin expression

**Figure 6:**
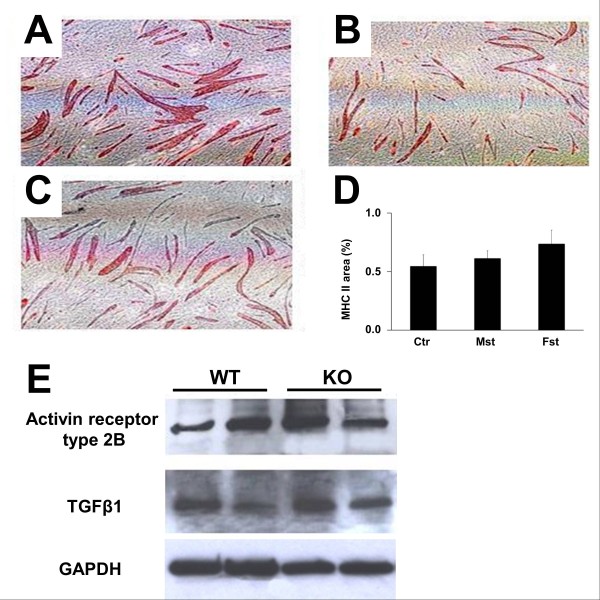
**Myostatin and follistatin fail to modulate the myogenic differentiation of MDSCs, although the myostatin receptor is expressed**. **(A-D) **Confluent WT MDSCs in myogenic medium were incubated in triplicate on six-well plates for 1 week with recombinant myostatin **(B) **or follistatin **(C) **or with no addition **(A)**, and subjected to immunocytochemistry for MHC II (40×). The relative area occupied by the MHC II^+ ^myotubes was estimated by quantitative image analysis (15 fields/well/three wells) **(D)**. Cont, control; Mst, myostatin; Fst, follistatin. No myotubes were formed in confluent Mst KO under any treatment (not shown). **(E) **Western blot detection in confluent MDSCs from both mice strains of the expression of the ActRIIb and TGF-β1, in two successive passages for each cell line. Myostatin was not detected. MDSCs, muscle-derived stem cells; WT, wild type; Mst KO, myostatin knockout; MHC, myosin heavy chain.

Incubation of Mst KO MDSCs for 3 days with 5-azacytidine, a demethylating agent and potent inducer of myogenic capacity in pluripotent cell lines [11,14], before their reaching confluency and switching to myogenic medium, failed to induce myotube formation, but it also failed to stimulate it in the WT MDSCs (not shown). Follistatin, which should upregulate myotube formation by binding myostatin, was also virtually ineffective on WT MDSCs, and the same resistance to modulation was observed under recombinant myostatin, which should exert the opposite effects. Figure 6A through D shows that the area occupied by MHC II^+ ^myotubes was not reduced in the cultures treated from the start of myotube induction with 2 μg/ml myostatin (Figure 6B), or increased by 0.5 μg/ml follistatin (Figure 6C), as compared with untreated controls (Figure 6A). Changes were not significant (Figure 6D). This failure of myostatin and follistatin to affect myogenesis in any type of MDSCs occurred despite these cells expressing the myostatin receptor ActRIIb in both cultures as shown by Western blot (Figure 6E), implying that they should be responsive to exogenous myostatin. Endogenous myostatin expression was not detected in any untreated culture (not shown), even if TGF-β, another key member of the TGF-β family, was expressed (Figure 6E). Finally, neither the monoclonal nor the polyclonal antibodies against myostatin affected myogenesis in the WT MDSCs, as compared with the respective cultures incubated with control IgG (not shown).

This suggests that the WT MDSC ability to form myotubes is refractory to the modulation by myostatin, and this was confirmed by transfection with the AdV Mst cDNA construct, or alternatively, with the AdV Mst shRNA, which also expresses β-galactosidase, which did not inhibit or stimulate this process, although myostatin and β-galactosidase were respectively expressed (not shown). The suppression of myotube formation in the Mst KO MDSCs by myostatin genetic inactivation and the lack of response to demethylating agents suggests that this is a complex imprinting process occurring during their embryologic generation, of a different nature than the resistance to paracrine and autocrine myostatin modulators observed in the WT MDSCs.

### Mst KO MDSCs stimulate myofiber repair in the injured gastrocnemius of the aged mdx mouse, but the absence of myostatin in these cells does not confer on them a distinctive advantage over the WT MDSCs

**Figure 7:**
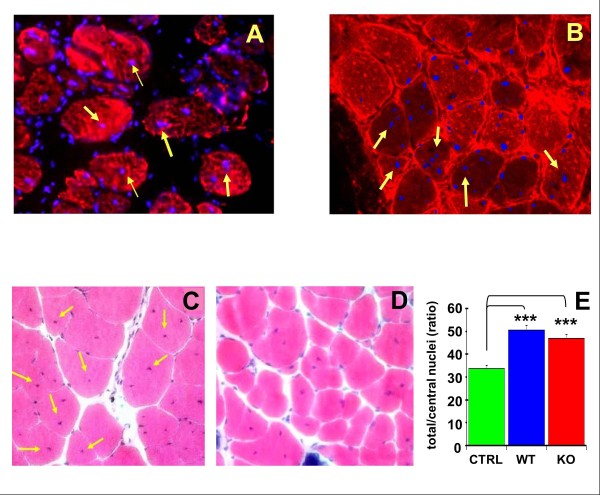
**Mst KO MDSCs failed to generate myotubes *in vitro*, but *in vivo *stimulate tissue repair comparable to the WT MDSCs**. Aged (10-month-old) mdx mice were used to maximize myofiber loss and lipofibrotic degeneration in the gastrocnemius. **(A) **Muscles were cryoinjured, implanted with 0.5 × 10^6 ^DAPI-labeled WT MDSCs, and allowed to undergo repair for 10 days. Frozen muscle sections were stained for MHC-II with Texas red streptavidin, and merging of blue and red fluorescence was obtained (200×). MDSC nuclei centrally located within myofibers are indicated with yellow arrows. **(B**) Similar picture, but for Mst KO MDSCs. **(C) **Gastrocnemius injury in the aged mdx mice was performed in the two apexes of the muscle with notexin, and muscles were injected 4 days later with saline or with 1.0 × 10^6 ^WT MDSCs or **(D) **Mst KO MDSCs in saline (*n *= 5/group). Repair was allowed to proceed for 3 weeks. Hematoxylin/eosin staining was performed in frozen sections, and a representative picture for each case shows myofibers from the gastrocnemius implanted with MDSCs, with arrows pointing to abundant central nuclei (200×). **(E**) Quantitative image analysis of these tissue sections (WT), in comparison to tissue sections from Mst KO MDSC-implanted mice (KO) and saline-injected controls, based on 12 fields per section, three sections per animal. ***P < 0.001. Mst KO, myostatin knockout; MDSC, muscle-derived stem cell; WT, wild type; mdx, X chromosome-linked muscular dystrophy; DAPI, 4', 6-diaminido-phenylindole; MHC, myosin heavy chain; KO, knockout.

To test the persistence of MDSCs after implantation into the muscle, DAPI-labeled cells were implanted into the cryolacerated gastrocnemius of the aged mdx mouse, and frozen tissue was examined with immunocytofluorescence for MHC II after 2 weeks. Figure 7A shows that the blue fluorescent WT MDSC nuclei are detected in many of the red fluorescent myofibers, and many of these nuclei are central, as may be expected from regenerating myofibers (yellow arrows). Other nuclei are seen in the interspersed connective tissue among the fibers. The Mst KO MDSCs acted similarly (Figure 7B). Although DAPI nuclear labeling of implanted cells may be prone to fading after long periods of implantation, it was adequate at 2 weeks to trace MDSC uptake and survival. However, the overlapping is only suggestive and cannot conclusively show MDSC conversion into myofibers. The MDSC implantation was then repeated into the notexin-injured muscle of aged mdx mice, by using either WT or Mst KO cells, or vehicle, and killing at 3 weeks for measuring myofiber repair. Panels C and D show representative muscle tissue sections stained with hematoxylin-eosin (HE) from mice injected with WT MDSCs and Mst KO MDSCs, respectively, where the central regenerating nuclei are visible. When the central nuclei were counted by quantitative image analysis, WT MDSCs significantly stimulated by 54.5% the appearance of central nuclei on hematoxylin/eosin-stained frozen tissue sections in comparison to control injured muscle receiving vehicle. The Mst KO MDSCs that had failed to convert into myotubes *in vitro *were now able *in vivo *to increase significantly by 42.4% the number of central nuclei in the myofibers in comparison to the vehicle-injected mice (Figure 7D). However, this stimulation of myofiber repair did not surpass the efficacy of the WT MDSCs, contrary to what was originally expected from the absence of myostatin in the Mst KO MDSCs.

**Figure 8:**
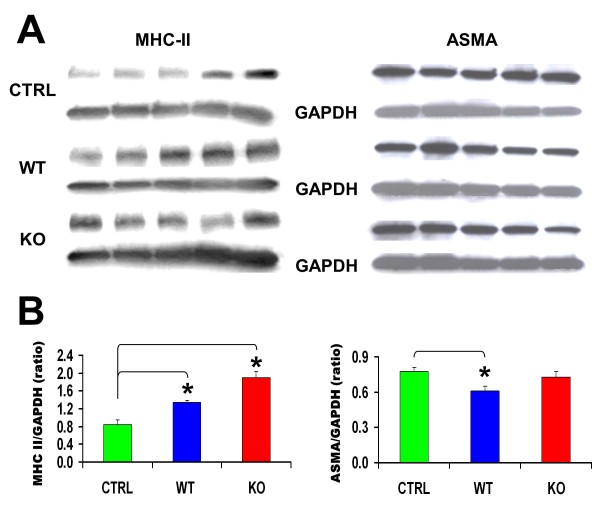
**Implanted Mst KO MDSCs stimulate more effectively than do WT MDSCs the expression of MHC-II in the muscle, but do not reduce ASMA**. **(A) **Western blot analysis for MHC II, ASMA, and GAPDH (reference gene) in homogenates of skeletal muscle tissue from the central region adjacent to area examined histochemically in Figure 7C. Each lane corresponds to an individual mouse homogenate (*n *= 5/group), and the three gels were run simultaneously. **(B) **Densitometric evaluation of the relative intensity expressed as ratios of the MCH-II or ASMA and GAPDH bands. *P < 0.05. Mst KO, myostatin knockout; MDSC, muscle-derived stem cell; WT, wild type; MHC, myosin heavy chain; ASMA, α-smooth muscle actin; GAPDH, glyceraldehyde 3-phosphate dehydrogenase.

These results were supported by the fact that Mst KO MDSCs significantly increased the expression of MCH-II in the notexin-injured mdx aged muscle estimated by Western blot, as compared with the vehicle-injected muscle, and this was slightly more effective than WT MDSC (Figure 8, left). It should be emphasized that this measurement was conducted in the central region of the muscle, distant from the notexin-injured sites at both ends of the muscle used for the tissue-section studies, suggesting that the stimulatory effect on MHC-II expression by MDSCs may have been even higher in the injured tissue. However, Mst KO MDSCs did not reduce ASMA expression, an indicator of myofibroblast generation, and hence fibrosis, whereas the WT MDSCs did decrease this expression by 23% (right).

**Figure 9:**
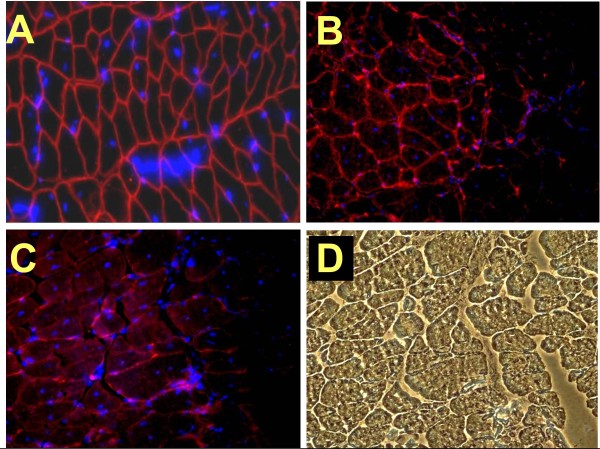
**The dystrophin^+ ^MDSCs restore some dystrophin expression in the injured mdx gastrocnemius**. **(A) **Myofibers from the intact gastrocnemius from the WT mouse, the source of WT MDSCs, show in a dual immunofluorescence merge all myofibers stained for dystrophin, and nuclei stained with DAPI (Vectashield mounting medium; Vector Laboratories, Burlingame, CA, USA) (200×). **(B) **In other tissue sections, DAPI-labeled implanted Mst KO MDSCs appear to have fused with the mdx myofibers, showing dystrophin^+ ^staining in a small area. **(C) **A similar picture but with WT MDSCs. **(D) **The same field as in (C), examined under visible light, confirming the integrity of the myofibers, including the dystrophin^- ^area. MDSC, muscle-derived stem cell; mdx, X chromosome-linked muscular dystrophy; WT, wild type; DAPI, 4', 6-diaminido-phenylindole; Mst KO, myostatin knockout.

Untreated WT mice skeletal muscles show dystrophin expression in frozen sections, as evidenced by the sarcolemma immunofluorescence around the myofibers (Figure 9A), a gene that is carried by their respective MDSCs. The nuclei here were detected by direct DAPI labeling of the tissue sections. In the case of the mdx mice that were implanted with DAPI-labeled WT MDSCs (Figure 9C) or Mst KO MDSCs (Figure 9D), some of the myofibers, which in the mdx muscle are negative for dystrophin, showed a partial dystrophin^+ ^staining of the sarcolemma in one of the areas of some sections. Others remained dystrophin negative, as evidenced by comparison of the same area visualized for dual fluorescence (Figure 9C) or with light microscopy (Figure 9D). The overlapping of DAPI-labeled nuclei and dystrophin^+ ^myofibers suggests that, as in the case of Figure 7, some conversion or fusion of the implanted MDSCs into myofibers occurs, but that this process may be much less frequent than the stimulation of endogenous satellite cells or stem cell differentiation or fusion, or the spontaneous myofiber reversion.

**Figure 10:**
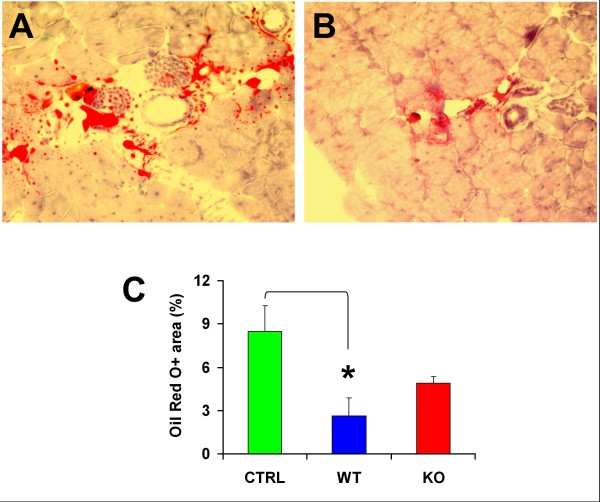
**The Mst KO MDSCs are less effective than the WT MDSCs in reducing fat deposits in the injured mdx mouse gastrocnemius**. **(A) **Representative picture of a positive field from frozen-tissue sections from the untreated mdx-injured gastrocnemius, adjacent to those shown in Figure 8D, fixed in formalin and stained with Oil Red O, showing mostly interstitial fat and occasional myofiber fat infiltration (200×). **(B) **Staining of a representative field from sections from the muscle implanted with WT MDSCs; the Mst KO pictures were similar, but the reduction in staining was less marked. **(C) **Quantitative image analysis of the tissue sections from the three rat groups, based on 12 fields per tissue section and the total positive area per section (percentage), calculated as a mean for three adjacent sections per rat, and five mdx mice/group. *P < 0.05. Mst KO, myostatin knockout; MDSC, muscle-derived stem cell; WT, wild type; mdx, X chromosome-linked muscular dystrophy.

As expected, fat infiltration is visible in the injured aged gastrocnemius from vehicle-injected aged mdx mice, mainly interstitially, but also as Oil Red O^+ ^small regions around or inside myofibers (Figure 10A and 10B). WT MDSCs were effective in significantly reducing this fat infiltration by 68%, and Mst KO MDSCs also induced a decrease, although it was not significant (Figure 10C).

## Discussion

To our knowledge, this is the first report testing the myogenic capacity of MDSCs isolated from transgenic mice with inactivation of the myostatin gene, in comparison to the WT MDSC, both *in vitro *and in the injured muscle of the aged mdx mice *in vivo*. Our main findings were (a) in contrast to WT MDSCs, Mst KO MDSCs were unable to form myotubes *in vitro*, although no major differences were found between both MDSC cultures in terms of morphology, replication rates, expression of most members of a subset of key embryonic-like stem cell and other markers, and nonmyogenic multilineage differentiation; (b) however, a fundamental difference is that the expression of key genes in myogenesis seen in WT MDSCs such as *actc1, acta1*, and *myoD*, was virtually obliterated in Mst KO; (c) surprisingly, both types of MDSCs were refractory *in vitro *to the modulation or induction of myotube formation by well-known regulators of this process, or of myofiber number *in vivo*, such as demethylating agents, myostatin inhibition or overexpression, or follistatin, although myostatin receptors are expressed in MDSC cultures; (d) the myofiber regeneration and anti-lipofibrotic capacities of WT MDSCs were evident even in the environment of a severely injured mdx gastrocnemius at an age at which lipofibrotic degeneration is considerable; (e) in turn, these capacities, blocked in cell culture, were recovered in Mst KO MDSCs when they were implanted in the injured mdx aged-muscle setting, even if not at the level expected from the supposed paracrine effects triggered in the MDSCs by the absence of myostatin.

It should be noted that although notexin-induced injury is not clinically relevant for DMD, it is experimentally convenient by stimulating cell engraftment on implantation and also inducing more lipofibrotic degeneration both in mdx and Mst KO mice [56,57], thus providing an adequate environment for testing the MDSC-repair effects. The high variability in the repair response that is often associated with notexin injection was not observed in the current work.

The WT MDSC used here as control, fulfill all the criteria that have been extensively defined as potential tools for skeletal muscle, cardiac, and osteogenic repair on implantation into the target organs [29,34]. In the current work, MDSCs were isolated as the pP6 fraction by using a modification of the extensively validated preplating procedure on collagen-coated flasks and Sca1 selection, and shown to have the expected morphology, rapid replication for at least 50 passages, express MDSC markers such as Sca1, CD44, and CD34, and the stem cell gene Oct 4, and the ability to differentiate *in vitro *into multiple cell lineages. The latter capability includes a robust formation of multinucleated and branched myotubes that is assumed to translate *in vivo *into their ability to donate their nuclei to injured skeletal myofibers or most likely to stimulate paracrinely their regeneration through paracrine trophic effects [32-34]. This is evidenced by a much higher number of centrally located nuclei, and even some central location of the DAPI-labeled implanted nuclei. In previous studies, we showed that WT MDSC generate at least smooth muscle and epithelial cells when implanted into urogenital tissues [27,28], adding to the extensive demonstration of their stem cell nature [7,12,26,58] related to their putative origin as myoendothelial stem cells in the muscle and other tissues [59].

Another novel finding here is that WT MDSCs have some embryonic-like stem cell features, mainly the expression of nuclear *Oct 4 A, myc, LIF*, and other embryonic stem cell genes. *Oct 4 *is a key not only for embryonic stem cell programming, but also for iPS generation, where it can act virtually by itself [60]. Our MDSC cultures contain some tiny rounded cells similar to the very small embryonic-like stem cells (VSELs) described in many adult organs [61], and other larger ones.

An important finding is the unexpected observation that myotube formation by the WT MDSCs *in vitro *is refractory to modulation by agents that are well known to affect this process, or skeletal muscle mass *in vivo*. The fact that myotube formation by WT MDSCs was not influenced by (a) demethylating agents like azacytidine that stimulate 'stemness" in cell lines [51]; (b) downregulation or overexpression of myostatin, despite the detectable expression of its receptor (ActIIb); (c) counteracting myostatin activity by the respective antibodies or follistatin, that *in vivo *stimulate myofiber growth [17,19,20]; poses questions related to the role of MDSCs during normal myogenesis. A study showing that myostatin stimulated fibroblast proliferation *in vitro *and induced its differentiation into myofibroblasts, while increasing TGF-β1 expression in C2C12 myoblasts, did not examine MDSC differentiation [12]. The claim of a small inhibitory effect of myostatin on the fusion index in MDSCs [58] may indicate less fusion efficiency but might not entirely reflect the actual effects on the number and size of myotubes, as determined here. This question requires further clarification in terms of the actual modulation of MDSC differentiation.

It may be speculated that satellite cells rather than MDSCs are the only myogenic progenitors during normal myofiber growth, as opposed to repair of damaged fibers [62]. Therefore the selected *in vitro *conditions may not mimic the repair process, or alternatively, unknown *in vivo *paracrine or juxtacrine modulators may modify the response of MDSCs to the better-characterized agents tested in this work. Another possibility is that myostatin and other modulators investigated here would stimulate *in vivo *satellite cell replication and fusion to the adjacent myofibers to induce hypertrophy, without truly affecting MDSC differentiation or fusion.

We are unaware of any report on the isolation or characterization of MDSCs from the Mst KO. Therefore, it is also both novel and unexpected to find that these cells obtained from the same skeletal muscles as the WT MDSCs, by using identical procedures, and displaying rather similar nonmyogenic pluripotency and stem cell-marker features, are however completely unable to form myotubes *in vitro*. In fact, our prediction was that the Mst KO MDSCs should be more myogenic than the WT MDSCs because of the absence of the myogenic inhibitor myostatin, The fact that Mst replenishment, either as recombinant protein or as cDNA, does not counteract the unexpected myogenic blockade found in the Mst KO MDSCs, suggests speculatively that these cells have been imprinted in the embryo by the myostatin genetic inactivation through downstream pathways that have become unresponsive to the *in vitro *myostatin modulation that we explored here. This may involve genes in other myogenic pathways whose expression may be altered, as we observed in Mst KO MDSCs. However, validation of this assumption requires further investigation.

An interesting corollary is the activation of the *in vitro*-suppressed myogenesis in Mst KO MDSCs, and/or their ability to fuse with preexisting myofibers, after their implantation into the notexin-injured mdx gastrocnemius. At the age selected (10 months), this muscle experiences the considerable damage that occurs in the diaphragm much earlier [3,4], and this is compounded by injury. It may be speculated that the restoration of myotube (myofiber) formation by Mst KO MDSCs in this setting occurs by paracrine or juxtacrine modulation, possibly of some of the key genes silenced in these cells. Estimation of their products and proof-of-function approaches may elucidate this issue. The fact that although Mst KO MDSCs are able to fuse with or differentiate into new myofibers, they do not increase the muscle-repair process in a clearly more efficient way than do WT MDSCs, may possibly result from the persistent myostatin expression in the fibers that may counteract its absence in Mst KO MDSCs. This suggests the need to block myostatin systemically in the host muscle [63,64], not just in the implanted MDSCs, and our findings do not contradict the potential use of this approach

One of the genes that may be involved in the silencing of Mst KO MDSC myogenesis *in vitro *and its reactivation *in vivo *is the cardiac α-actin (*Actc*), the major striated actin in fetal skeletal muscle and in adult cardiomyocytes, but strongly downregulated in adult skeletal muscle to 5% of the total striated actin [65], and whose mRNA is highly expressed in the proliferating (nondifferentiating) WT MDSCs but at very low level in the Mst KO MDSCs. The same applies to the α1-actin (*Acta1*) mRNA, the adult protein encoding thin filaments [66]. Because actins are so crucial for cell division, motility, cytoskeleton, and contraction, and mutations are associated with severe myopathies, it would not be surprising that their downregulation could cause the lack of myogenic commitment *in vitro *in Mst KO.

Similarly, the striking transcriptional downregulation of *myoD*, a critical early gene in skeletal myogenesis [67], confirmed at the protein level, and of secreted phosphoprotein 1, or osteopontin, a gene mostly involved in ossification, inflammation, and fibrosis, but postulated recently to participate in early myogenesis and skeletal muscle regeneration [68], may also trigger the absence of myogenic capacity in Mst KO. Interestingly, the fact that *Pax 3 *mRNA, upstream of MyoD in the myogenic signaling [69] is expressed in Mst KO MDSCs at higher levels than in WT MDSCs, suggests that the myogenic commitment of Mst KO and mdx MDSC is arrested at some point between these genes. Because a critical regulator of skeletal muscle development, *Mef2a *(myocyte enhancer factor 2a) [70], is expressed similarly in both MDSCs (as is *Pax 3*), albeit at very low levels, the silencing may occur at the level of the satellite cell marker, *Pax 7*. Therefore, it is not surprising that expression of a member of the cadherin family (cadherin-15) that is involved in later stages, such as myoblast differentiation and fusion [71], is so downregulated in Mst KO MDSCs.

## Conclusions

Our results show that MDSCs obtained from wild-type and Mst KO mice lacking myostatin express *Oct 4 *and other embryonic-like stem cell genes and appear similar in most features, except for the null or poor expression in Mst KO MDSCs of some critical early genes. These genes encode factors critical for myogenesis and for maintaining the integrity of myotubes and myofibers, thus possibly leading to their inability to form myotubes *in vitro*. The cross-talk of Mst KO MDSCs with myofibers and other cell types in the host injured mdx muscle may release the pertinent gene silencing and restore the typical myogenic ability of the MDSCs. Although our results do not prove the initial working hypothesis that myostatin inactivation would enhance the myogenic capacity of MDSCs, this possibility still needs further *in vivo *testing by blocking myostatin, not just in the implanted MDSCs, but also in the host muscle with follistatin, shRNA, antibodies, or other procedures. Finally, systemic muscle-targeted WT MDSC implantation that was previously shown as a promising approach to stimulate repair in the adult dystrophic muscle [5,12,45,46], may even be effective in the setting of an injured aged dystrophic skeletal muscle with severe bouts of necrosis [4].

## Abbreviations

AdV-CMV-Mst375: adenovirus construct expressing the mouse myostatin full-length cDNA under the CMV promoter; AdV-Mst shRNA: shRNA against myostatin RNA; ASMA: α-smooth muscle actin; DMD: Duchenne muscular dystrophy; MDSC: muscle-derived stem cell; Mst KO: myostatin knockout mouse; QIA: quantitative image analysis; TGF-β1: transforming growth factor β1; VSEL: very small embryonic-like stem cell; WT: wild-type mouse.

## Competing interests

The authors declare that they have no competing interests.

## Authors' contributions

NGC conceived and designed the study; JT, DV, RG, IK, GN, and KWB performed and executed the experiments; NGC prepared the manuscript. All authors read, revised, and approved the final manuscript.
